# Retraction: *Eucommia ulmoides* Oliver Extract, Aucubin, and Geniposide Enhance Lysosomal Activity to Regulate ER Stress and Hepatic Lipid Accumulation

**DOI:** 10.1371/journal.pone.0328095

**Published:** 2025-07-11

**Authors:** The PLOS One Editors

After this article [1] was published, the corresponding author contacted PLOS requesting retraction of [1] due to concerns raised regarding results presented in Figures 1–4 and Figures 6–7.

Specifically:

- In multiple panels, two or more lanes within a single western blot panel appear similar to each other, including:
  - In Fig 1B, p-PERK, p-elF-2α, elF-2α, ATF-6α, IRE1-α and β-actin
  - In Fig 2B, Cell lysate ApoA1, Medium ApoA1, and Medium CBB
  - In Fig 3, GRP78, p-PERK, CHOP, elF-2α, ATF-6α, and IRE1-α
  - In Fig 4B, Medium ApoB and Medium CBB
  - In Fig 6A, PERK, p-PERK, IRE-1α, and CHOP
  - In Fig 6C CBB
  - In Fig 7A, GRP78 and p-elF-2α
  - Fig 7E, Liver ApoA1
- There appears to be partial overlap between panels presented in the following figures:
  - Multiple panels in Fig 1B and multiple panels in Fig 3
  - Multiple panels in Fig 2B and multiple panels in Fig 4B
  - Fig 6A β-actin and Fig 6C β-actin
  - Fig 6C Medium CBB and Fig 7E Plasma CBB
  - Fig 7A β-actin and Fig 7E Liver β-actin
- There appear to be vertical discontinuities in the following panels:
  - Fig 1A CHOP
  - Fig 7A p-PERK
  - Fig 7A IRE1α
- The Fig 2A Con and Pal panels in this article [1] appear similar to the Fig 2A Con and Pal lower panels in [2].

The corresponding author stated that the original underlying data are no longer available. They stated that the above issues arose due to multiple researchers working on related materials in a shared file location. The corresponding author stated that in light of the issues in [1], they are repeating the experiments and provided some of the repeat data to PLOS. In the absence of the original data, PLOS does not consider that repeat data are sufficient to address the concerns raised with the figures as published.

In light of the above concerns, which raise concerns about the reliability and integrity of the published results, the *PLOS One* Editors retract this article.

HYL, GHL, MRL, NYK, SHK, and HJC agreed with the retraction. HKK, YCL, and HRK either did not respond directly or could not be reached.
